# Scorpion sting and allergic reaction to scorpion venom: A case-based review 

**DOI:** 10.5414/ALX400582

**Published:** 2024-06-25

**Authors:** Jozélio Freire de Carvalho

**Affiliations:** Núcleo de Pesquisa em Doenças Crônicas não Transmissíveis (NUPEN), School of Nutrition from the Federal University of Bahia, Salvador, Bahia, Brazile

**Keywords:** scorpion, scorpionism, envenomation, urticaria, allergy

## Abstract

Objective: To describe a young patient with scorpion sting (SS) with typical lesions of urticaria besides the local SS clinical picture. Materials and methods: A systematic screening of articles dating from 1966 to 2021 was conducted in the main databases. All articles included the association between SS and urticaria. A new case report is added to the published list. Results: The literature search found 5 articles with 29 patients with SS and urticaria/allergic reactions. We performed our analysis by adding our present case, resulting in a total of 30 cases. Most were male, and their ages varied from 29 to 48 years. Regarding SS severity, most were mild or moderate. In two articles, patients had more than one sting. The allergic reaction varied from urticaria, pruritus, flushing, angioedema, wheezing, rhinorrhea, sneezing, consciousness alterations, and gastrointestinal and cardiovascular alterations. In 5/6 (83%) articles, the patients were alive at the study time. One subject died from anaphylactic shock. Conclusion: The present article systematically reviewed all published cases of SS and allergic reactions to scorpion venom. It is an infrequent association; most patients are male and in the productive age, and reaction may vary from mild to severe, including death.

## Introduction 

Scorpion envenomation is quite common worldwide, mainly in tropical and subtropical countries. The incidence and severity of envenomation are higher in seven world areas: the northern Sahara, the southern and eastern regions of Africa; the Middle East, south India, Mexico, Brazil, and the Amazonian basin area (Guyanas, Venezuela, and northern Brazil). In Brazil, 156,833 cases of scorpionism with 94 deaths were reported in 2018 by DATATOX, a nationwide database on toxicology, including animals [1]. The clinical picture of scorpion sting (SS) varies from a mild picture with local pain and localized signs like sweating and paresthesia to moderate and severe, with systemic manifestations such as nausea, vomiting, abdominal pain, heart failure, and acute pulmonary edema. The intensity of these symptoms is classified into moderate or severe cases. The treatment involves analgesia and local anesthesia for pain and antivenom serotherapy in moderate and severe cases, with intensive care unit monitoring [2]. 

There are several descriptions of urticaria and anaphylaxis due to serotherapy [3]. A few reports on urticaria and anaphylaxis are secondary to the scorpion venom itself [4, 5, 6, 7, 8]. 

This article aims to perform a case-based review of the literature on this rare association between SS and urticaria, adding a new case report that was successfully treated with antihistamine and glucocorticoid. 

## Materials and methods 

### Patient selection 

The present research is characterized as a descriptive and cross-transversal study with a retrospective analysis of the medical records of a patient diagnosed with both SS and urticaria or anaphylaxis. Informed consent was obtained from the patient’s relatives to publish her case. 

### Literature review 

A systematic screening of articles published in PubMed/MEDLINE, LILACS, SciELO, Scopus, Web of Science, and Cochrane dating from 1966 to February 2021 was performed. The following terms were used: “scorpion” or “scorpionism” and “urticaria” or “allergy” or “anaphylaxis.” No language restrictions were used. Also, a new case report with the association above is described in detail. The author independently reviewed the articles. The following information was collected: demographic characteristics (sex, age), clinical and laboratory features (clinical presentation, autoantibodies, the onset of symptoms and progression), therapy provided, and response to reported treatment. A thorough analysis of these 5 scientific articles and their list of references was conducted. Duplicate articles, insufficiently detailed or not informative enough, were excluded. 

### Case report 

A previously healthy 19-year-old woman was bitten by a scorpion on her arm. She experienced mild local pain and paresthesia. After 2 hours, she developed wheals in both upper limbs with moderate pruritus. She was treated with diphenhydramine and intravenous methylprednisolone 100 mg with progressive improvement. No anti-scorpion venom was needed. The specimen was identified by an entomologist as Tytius confluens. The patient stayed in the hospital for 1 day and was utterly asymptomatic upon discharge. 

## Results 

Table 1 summarizes the patients who reported this rare association between SS and urticaria/anaphylaxis. The literature search found 5 articles [4, 5, 7, 8] with 29 patients. Therefore, we performed our analysis by adding our present case, resulting in a total of 30 cases. 

The articles came from the United States (n = 3), Brazil (n = 2), and Algeria (n = 1). 

17 patients were male, and 9 were female; in the other cases, the article did not describe the gender. Age varied from 29 to 48 years old. Regarding SS severity, most cases were mild or moderate. In 2 articles, patients had more than one sting. 

The allergic reaction varied from urticaria, pruritus, flushing, angioedema, wheezing, rhinorrhea, sneezing, consciousness alterations, and gastrointestinal and cardiovascular alterations. The time between the ﻿tings and onset of symptoms varied from 1 minute to 2 hours. 

In 5/6 (83%) articles, the patients were alive at the study time; 1 subject died from anaphylactic shock. 

## Discussion 

We systematically reviewed the published articles on patients with SS who developed allergic reactions, including urticaria and anaphylaxis. In addition, we described for the first time a patient with *T. confluens* envenomation who developed urticaria. 

The strength of our article is the inclusion of all articles that reported an allergic reaction (urticaria and anaphylaxis) to the scorpion venom described in the conventional databases. In addition, all envenomation cases were confirmed by entomologists. 

Besides the well-known toxicity reactions to scorpion venoms, some patients may also have immunologically mediated responses characterized by an allergy that varies from mild to severe (9 anaphylactic reactions). Moreover, there is a description of an allergic reaction after the first sting, which suggests the existence of IgE sensitization to allergens in scorpion venom, as observed in our patient [6]. One possible explanation would be the cross-reactivity between scorpion venom and ant venom. This was studied in 9 patients with a previous history of ant allergy, and the authors found positivity in the skin test in 6/9 (67%) for scorpion venom [5]. 

It is known from animal models that scorpion toxin has immunological capabilities [9]. Therefore, in this line, urticaria may appear after contact with scorpion toxins [4, 5, 6, 7, 8]. 

Scorpion toxins are a mixture of proteins, peptides, nucleotides, and amines. They target excitable and immunological cells, especially potassium, calcium, chlorine, and sodium channels. An increasing number of studies have focused on their composition and bioactivity, and these toxins vary between the scorpion species [10]. 

In the More et al. study [4], the authors identified antigens from scorpion toxins from *Centruroides vittatus* and evaluated the immediate hypersensitivity to the venom. They found that 4 out of 5 patients had positive skin test reactions. In all patients, gel electrophoresis demonstrated multiple proteins; most were identified as allergens on IgE immunoblots, and the enzymatic activity was found to include phospholipase A, alkaline phosphatase, esterase, esterase lipase, and acid phosphatase [4]. 

An essential topic for clinicians is the distinction between SS toxicity and hypersensitivity. The clinical history and physical examination are the basis for that. In SS toxicity, local signs and symptoms are predominantly characterized by pain and paresthesias with mild or absent edema. In children younger than 5 years old, SS envenomation may lead to severe cases of heart failure [2]. On the other hand, SS hypersensitivity will be observed in skin lesions compatible with urticaria, allergic reactions, pruritus, and even severe manifestations of angioedema and cardiovascular involvement, according to Ring and Messmer’s anaphylactic classification [11]. 

This study has some limitations: First, cases from grey literature may not have been found in our literature search; second, in two articles, the main objective was to describe laboratory or in vivo tests for allergic hypersensitivity, not the clinical pictures. 

In conclusion, the present study reports a rare manifestation of urticaria in a patient with SS. It highlights another possibility besides SS or scorpion envenomation, which is the development of allergic reactions to the toxins of this insect. Conventional therapy using antihistamines and glucocorticoids is recommended. 

## Funding 

None. 

## Conflict of interest 

None. 

**Table 1.** All published cases of scorpionism and associated urticaria/allergy reactions.

**Author, year [reference]**	**Country**	**Number of subjects**	**Age (years), sex**	**Scorpion sting**	**Scorpion specimen**	**Previous episodes of scorpion sting**	**Ring and Messmer anaphylactic classification [** 11 **]**	**Treatment**	**Time between sting and symptoms**	**Outcome**
Carvalho et al. 2021 (present case)	Brazil	1	22, female	Moderate	*Tytius confluens*	No	Grade I (urticaria over the trunk and upper limbs)	Anti-histamine plus glucocorticoid	2 hours	Alive
Melo et al. 2019 [8]	Brazil	1	44, male	Possibly severe	*Jaguajir rochae*	No	Grade III (anaphylactic shock)	Patient was dead upon arrival at the emergency department	Minutes	Death
More et al. 2004 [4]	United States	11	48 ± 8.8, 64% female	Mild to severe	*Centruroides vittatus*	One case	Grade III (urticaria, pruritus, flushing, angioedema, wheezing, rhinorrhea, sneezing, consciousness alterations, gastrointestinal and cardiovascular alterations)	ND	ND	Alive
Nugent et al. 2004 [5]	United States	4	ND	ND	*Centruroides vittatus*	ND	ND	ND	ND	Alive
Leynadier et al. 1997 [7]	Algeria	10	29.3 ± 21, 90% male	90% mild, 10% moderate	*Androctonus australis Hector*	Yes, varying from 2 to more than 8 times	ND	ND	ND	Alive
Demain et al. 1995 [6]	United States	3	38, 53, and 57; all-female	Moderate (n = 1) and severe (n = 2)	*Centruroides vittatus*	2 – 5 in all cases	Grade II	ND	1 – 2 minutes to 1 hour	Alive

ND = not described.
